# Synthesis, Physiochemical Properties, Photochemical Probe, and Antimicrobial Effects of Novel Norfloxacin Analogues

**DOI:** 10.5402/2011/184754

**Published:** 2011-03-06

**Authors:** Dina A. Bakhotmah, Reda M. Abdul-Rahman, Mohammad S. Makki, Mohamed A. El-Zahabi, Mansor Suliman

**Affiliations:** ^1^Joint Supervision Program, King Abdulaziz University, P.O. Box 80215, Jeddah 21589, Saudi Arabia; ^2^Department of Chemistry, Faculty of Sciences, King Abdul-Aziz University, P.O. Box 80203, Jeddah 21589, Saudi Arabia; ^3^Department of Organic Chemistry, Faculty of Pharmacy, King Abdul-Aziz University, Jeddah 21589, Saudi Arabia; ^4^Department of Pharmacology, Faculty of Medicine, King Abdul-Aziz University, Jeddah 21589, Saudi Arabia

## Abstract

The emerging resistance to antimicrobial drugs demands the synthesis of new remedies for microbial infections. Attempts have been made to prepare new compounds by modifications in the quinolone structure. An important method for the synthesis of new quinolone is using Vilsmeier approach but has its own limitations. The present work aimed to synthesize novel norfloxacin analogues using modified Vilsmeier approach and conduct preliminary investigations for the evaluation of their physicochemical properties, photochemical probe, and antimicrobial effects. In an effort to synthesize norfloxacin analogues, only 7-bromo-6-N-benzyl piperazinyl-4-oxoquinoline-3-carboxylic acid was isolated using Vilsmeier approach at high temperature, where *N*, *N*′-bis-(4-fluoro-3-nitrophenyl)-oxalamide and *N*, *N*′-bis-(3-chloro-4-fluorophenyl)-malonamide were obtained at low temperature. Correlation results showed that lipophilicity, molecular mass, and electronic factors might influence the activity. The synthesized compounds were evaluated for their antimicrobial effects against important pathogens, for their potential use in the inhibition of vitiligo.

## 1. Introduction

The structure activity relationship (SAR) for the quinolone skeleton 1-alkyl-1,4-dihydro-4-oxo-quinoline-3-carboxylic acid studies revealed that the 6-halogen atom, especially the 6-fluorine, is responsible for the potency as represented by the binding capacity with DNA gyrase and topoisomerase IV [1]. It is clear that chemical modifications at C-7 are suitable to control the pharmacokinetic properties and, hence, changes in the cell permeability of these antibiotics. *N*-piperazinyl derivatives of fluoroquinolones were introduced and demonstrated for various biological activities that possess broad-spectrum activity [2–6]. Furthermore, it is clear that the neutral species of fluoroquinolones are more lipophilic than the Zwitterionic form. Therefore, factors that can affect *N*-protonation like steric and electronic effect or charge density can also affect lipophilicity [7–9]. 

**Scheme 1:**
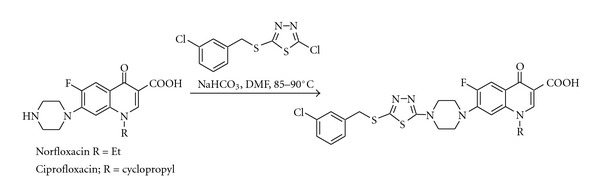
Norfloxacin modification.

Procopiou et al. [10] prepared a series of asymmetrical 1,4-disubstituted piperazines as a novel class of non-brain-penetrant histamine H3 receptor antagonists. In addition, Foroumadi et al. [11] synthesized a modified norfloxacin via heteroarylation of norfloxacin on *N*-piperazinyl position (Scheme 1). The antibacterial activity of these modified norfloxacin depends not only on the bicyclic heteroaromatic pharmacophore but also on the nature of the peripheral substitutions and their spatial relationship, such as solubility, thermal stability, hydrolysis, and a possibility to form a Zwitter ion. Meth-Cohn and Taylor [12] reported an important method for the synthesis of quinolones using reverse Vilsmeier approach but has its own limitations, like uncompleted cyclisation to the target quinolone. 

In the light of these observations, the aim of this work was to synthesize novel norfloxacin analogues using modified Vilsmeier approach and conduct preliminary investigations for the evaluation of their physicochemical properties, photochemical probe, and antimicrobial effects.

## 2. Materials and Methods

### 2.1. Equipment Used for the Characterization of the Produced Compounds

Electrothermal 9100 (fisher Scientific, US) was used to determine melting points or ranges. Infrared (IR) spectra were recorded on a Unicam Research Series 2000 FTIR. NMR spectra were recorded in DMSO or CDCl_3_ on a Bruker AVANCE 300 at 300 MHz. Mass spectrometry was performed on an Esquire 3000 plus, or Bruker ApexII, for low and high resolution. Elemental analysis was performed on an Exeter Analytical CE-440; GCMS was performed on Shimadzu GC-17A and QP-5000 Mass Spectrometer.

### 2.2. Materials Used for Microbiological Assay

Nutrient Agar, MacConkey Agar, Sabouraud Dextrose Agar, and dimethylformamide (DMF) were obtained from Sigma; Nalidixic acid (30 *μ*g/disk, Bioanalize, Egypt) and Nystain (manufactured by Pasteur Lab., Egypt, NS 100 units (100 *μ*g/disk) were used as reference antibiotics.

### 2.3. Synthesis of Norfloxacin Analogues

**Scheme 2:**
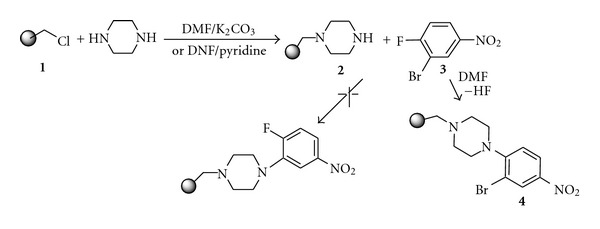


**Scheme 3:**
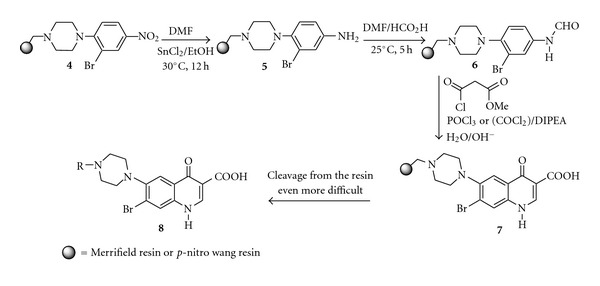


We used a solid phase via Merrifield resin through reactions of substituted piperazine with 3-bromo-4-fluoronitrobenzene. In the synthetic sequence, the Merrifield resin **(1) **was first suspended in dry DMF, and to this suspension was added an excess of piperazine (2-3 equivalents) in pyridine or anhydrous K_2_CO_3_ (6–8 equivalents). The reaction mixture was continued at 40°C for 24 hours then piperazine resin **(2)** was obtained, filtered, washed with CH_2_Cl_2_, and dried. Compound **2 **was resuspended in DMF and reacted with 3-bromo-4-fluoronitrobenzene **(3)** to give the 4-piperazine resin-supported-3-bromo-1-nitrobenzene **(4)** (not the expected 3-piperazine resin-supported-4-fluoro-1-nitrobenzene), (Scheme 2), which on reduction with SnCl_2_-EtOH yielded the 3-bromo-4-(4′-resin-supported benzyl piperazinyl)-1-aniline **(5)** and then by treatment with an excess of formic acid at room temperature for 12 hours produced the corresponded 3-bromo-4-(4′-resin-supported benzyl piperazinyl)-1-formanild** (6)**. The dry resin-supported formanilide** 6**, when reacted with Phosphorus oxychloride or Oxalyl chloride and methyl malonyl chloride **(7)** under reverse Vilsmeier conditions, mainly gave the resin-supported quinolone, 6-fluoro-7-piperazino-4-oxo-3-quinolone carboxylic acid, **(8) **(Scheme 3). The procedure, in general, yielded a mixture of by-products in low quantities, and TLC and GCMS were used for the assessment of the recovered cleavage products.

### 2.4. Preparation of 3-bromo-4-fluoronitrobenzene (**3**)

Equimolar mixture of nitric acid and sulphuric acid (1 : 1, 25 mL : 25 mL) was stirred at ~5°C. A solution of 2-fluorobromobenzene (25 g, 0.143 moL) in methanol (30 mL) was added to the mixture with gradual stirring over a period of 20–30 minutes. After complete addition, the temperature was raised gradually to 70°C for 1 h. After cooling, the reaction mixture was poured into cold water (20 mL), and the immediate cream solid precipitate was collected by filtration. Crystallization with CHCl_3_ gave a cream shiny crystals (29.23 g, 93% yield), mp 60–62°C (lit. [13] mp 58-59°C); *ν*_max⁡_/cm^−1^ 1535 and 1342 (NO_2_); *δ*_H_ (300 MHz; CDCl_3_) 7.29 (1H, t, *J* = 6.0 Hz, H-5), 8.24 (1H, m, H-6), 8.50 (1H, dd, *J* = 2.0 and 4.3 Hz, H-2); *δ*_C_ (75 MHz; CDCl_3_) 110.1 (d, *J* = 22.5 Hz, C-3), 117.1 (d, *J* = 22.5 Hz, C-5), 123.3 (d, *J* = 7.5 Hz, C-6), 129.6 (C-2), 144.4 (C-1), 162.9 (d, *J*_C−F_ = 195.7 Hz, C-4); *δ*F (MHz;CDCl3)-74.22 (s); *m*/*z* 221(M^+^, 44%), 219 (M^+^, 46%), 203 (3), 189 (17), 173 (38), 161 (14), 94 (M-Br-NO_2_, 100), 68 (25), 61 (7), 50 (38).

### 2.5. Preparation of 4-(4′-benzylpiperazin-1′-yl)-3-bromo-1-nitrobenzene (**9**)

Under dry conditions, 3-bromo-4-fluoronitrobenzene **(3)** (5.1 g, 23 mmoL) was dissolved in dry acetonitrile (2 mL), then anhydrous K_2_CO_3_ (9.6 g, 69.2 mmoL) was added followed by addition of *N-*benzylpiperazine (8 g, 46 mmoL) to the suspension mixture using a syringe; the temperature gradually raised to reflux for 12 h (or until the complete disappearance of the starting material). The reaction was monitored by TLC (CHCl_3_: petroleum ether (40–60), 50%). The acetonitrile was removed under* vacuo*, and the resulting solid was stirred in cold water (200 mL) for 20 minutes. The pale brown solid formed was recrystallized from CHCl_3_ to give bright yellow needle-like crystals of **9** (5.8 g, 81% yield), mp 123-124°C; [C_17_H_18_BrN_3_O_2_ Calc. C, 54.3; H, 4.8; N, 11.2. Found C: 54.5; H, 4.8; N, 11.1]; *ν*_max⁡_/cm^−1^ 1580, and 1339 (NO_2_); *δ*_H_ (300 MHz; CDCl_3_), 2.57 (4H, m, H-3′, and H-5′), 3.17 (4H, m, H-2′ and H-6′), 3.56 (2H, s, Ph-CH_2_), 7.12 (1H, d, *J* = 9.0 Hz, H-5), 7.24 (5H, m, Ph), 8.08 (1H, dd, *J* = 2.7 and 9.0 Hz, H-6), 8.26 (1H, d, *J* = 2.7 Hz, H-2); *δ*_C_ (75 MHz; CDCl_3_) 51.1 (C-3′ and C-5′), 52.8 (C-2′ and C-6′), 62.4 (CH_2_-Ph), 116.9 (C-3), 121.1 (C-5), 124.7 (C-6), 127.5 (C-2), 129.5 (Ph), 142.3 (C-1), 156.6 (C-4); *m*/*z* (M^+^373/375).

### 2.6. Preparation of 4-(4′-benzylpiperazin-1′-yl)-3-bromo-4-phenylamine (**10**) [14]

A pale yellow oil (2.7 g, 60% yield); *ν*_max⁡_ cm^−1^ 3150 (NH_2_); *δ*_H_ (300 MHz; CDCl_3_) 2.68 (4H, s, CH_2_-3′ and 5′) and 3.01 (4H, s, CH_2_-2′ and 6′), 3.57 (2H, s, Ph-CH_2_), 6.62 (1H, dd, *J* = 1.2 and 4.2 Hz, H-6), 6.94 (1H, d, *J* = 4.2 Hz, H-5), 6.97 (1H, d, *J* = 1.2 Hz, H-2), 7.37 (5H, m, Ph); *δ*_C_ (75 MHz; CDCl_3_) 52.2 (C-3′ and C-5′), 53.6 (C-2′ and C-6′), 63.3 (Ph-CH_2_), 114.9 (C-3), 120.1 (C-6), 121.1 (C-5), 121.8 (C-2), 128.4 (Ph), 142.3 (C-1), 143.4 (C-4); HRMS (ESI). Found: MH^+^, 346.0908. Calc. for C_17_H_20_BrN_3_: MH^+^ = 346.0919.

### 2.7. Preparation of 4-(4′-benzylpiperazin-1′-yl)-3-bromoformamide (**11**)

Formic acid (5 mL, 0.13 moL) was added to 4-(4′-benzylpiperazin-1′-yl)-3-bromo-4-phenylamine** (12) **(5 g, 14.4 mmoL), and the resulting clear solution was refluxed for 2 h. After cooling to room temperature, the reaction mixture was poured into ice water (10 mL), then NaHCO_3_  solution (10% w/v, 20 mL) was added gradually until no more effervescence (formation of neutral to slightly basic solution) was observed and the solution extracted with CH_2_Cl_2._ (3 × 20 mL). The organic layers were combined, washed with NaHCO_3 _solution (10%, 20 mL), and dried over MgSO_4_. The solvent was removed *in vacuo* until complete dryness to give **11** as a brown solid which was purified by column chromatography on silica, eluted with CHCl_3_ to give a white solid (2.94 g, 54%), mp 73-74°C; [C_18_H_20_BrN_3_O Calc. C, 57.76; H, 5.39; N, 11.23. Found: C, 57.79; H, 5.41; N, 11.23]; *ν*_max⁡_/cm^−1^ 3320 (br, NH), 1716 (NCHO); *δ*_H_ (300 MHz; CDCl_3_) 2.68 (4H, br s, CH_2_-3′ and 5′), 3.06 (4H, br s, CH_2_-2′ and 6′), 3.62 (2H, s, Ph-CH_2_), 7.34 (6H, m, Ph+H-5), 7.48 (1H, dd, *J* = 1.2 and 4.2 Hz, H-6 ), 7.81 (1H, d, *J* = 1.2, H-2 ), 8.34 (1H, s, CHO), 8.58 (1H, s, NH); *δ*_C_ (75 MHz; CDCl_3_) 51.7 (C-3′ and C-5′), 53.2 (C-2′ and C-6′), 63.2 (Ph-CH_2_), 119.3 (C-3), 120.1 (C-5), 121.0 (C-6), 125.5 (C-2), 129.4 (C-Ph), 132.5 and 132.8 (C-1), 147.7 and 148.5 (C-4), 158.9 and 162.5 (*N-*CHO).

### 2.8. Vilsmeier Reaction of 4-(4′-benzylpiperazin-1′-yl)-3-bromoformanilide (**9**) and Formation of Compound **12**

In dry atmosphere, a solution of 4-(4′-benzylpiperazin-1′-yl)-3-bromoformamide (**11)** (1 g, 2.7 mmoL) in POCl_3_ (5 mL) was stirred for 15 minutes at 25°C. A solution of methyl malonyl chloride (1.12 g, 8.5 mmoL) in POCl_3_ (2 mL) was gradually added to the reaction mixture through a syringe. After addition was complete, the oil bath temperature was gradually raised to 130–140°C, and the reaction was continued for 12 h. The excess POCl_3_ was removed *in vacuo,* and the cooled black residue was dissolved in diethyl ether (20 mL), poured into ice (50 mL), and vigorously stirred for 2 h. The resulting mixture was made basic by the addition of aq. NaOH solution (30%, 10 mL), refluxed for 2 h, and cooled for 12 h in fridge (<5°C). Column chromatography on the resulting black gum (CHCl_3_ : MeOH, 90 : 10) gave 6-(4′-benzylpiperazin-1′-yl)-7-bromo-4-oxo-1,4-dihydro-quinoline-3-carboxylic acid **(12)**. It was recrystallized from EtOH to produce a yellow solid as (0.1 g, 5% yield); mp 285-286°C; *ν*_max⁡_/cm^−1^ 3525 (carboxylic OH), 1699 (carboxylic C=O), 1611 (COO^−^ st as), 1462 (COO^−^ st sy); *δ*_H_ (600 MHz; DMSO-*d*_6_) 3.13 (8H, br s, piperazine), 4.19 (2H, s, Ph-CH_2_), 7.45 (5H, m, Ph), 7.83 (1H, s, H-8), 8.15 (1H, s, H-5), 8.87 (1H, s, H-2), 15.21 (1H, br s, NH); *δ*_C_ 51.21 (piperazine), 60.0 (CH_2_), 107.4 (C-3), 115.0 (C-8), 124.4 (C-5), 124.6 (C-7), 126.1 (C-10), 128.6 and 130.6 (Ph), 136.1 (C-9), 147.3 (C-2), 166.1 (C-6), 177.3 (CO_2_H), 206.5 (C=O); HRMS (ESI). Found: MH^+^, 442.0764. Calc. for C_17_H_20_BrN_3_ : MH^+^ = 442.0761.

### 2.9. Solid-Phase Synthesis with 4-fluoro-3-bromo-1-Nitrobenzene

#### 2.9.1. Loading the Piperazine to Merrifield Resin

**General Resin Preparation**

The Merrifield resin (**1)** (5 g) was a suspension in dry DMF (20 mL) for 6–12 h. The resin had a gel-like appearance double its original volume.

To the resin suspension, a molar excess of free piperazine (5 g), pyridine (2 mL), or K_2_CO_3_ (3 g), stirred at 80°C for 24 h. The cold resin was then filtered and washed with water (2 × 20 mL), MeOH (2 × 20 mL), and CH_2_Cl_2_ (2 × 10 mL), then dried *in vacuo* for a minimum of 24 h or until a constant weight was achieved (5.6 g); *ν*_max⁡_ /cm^−1^ 3441 (NH); (Found: C, 85.1; H, 10.4; N, 2.9%).

#### 2.9.2. Preparation of 3-bromo-4-(Resin-Supported benzylpiperazine)-1-nitrobenzene (**4**)

3-Bromo-4-fluoro-1-nitrobenzene **(3)** (2 g) was stirred in dry DMF (10 mL), and anhydrous K_2_CO_3_ (3 g) was added to the suspended piperazine-Merrifield resin** (2)** (3 g), and the reaction was continued at 50°C for 24 h. The cold resin was filtered, then washed with water (2 × 20 mL), MeOH (4 × 10 mL), and finally with CH_2_Cl_2_ (2 × 10 mL). The solid was dried under* vacuo* for 24 h or until constant weight (4.6 g); *ν*_max⁡_ /cm^−1^ 1509 and 1339 (NO_2_).

#### 2.9.3. Preparation of 3-bromo-4-(4′-Resin-Supported benzylpiperazino)-1-aniline (**5**)

3-Bromo-4-(4′-resin-supported benzylpiperazine)-1-nitrobenzene **4** (2 g) was suspended in dry DMF (10 mL) for 12 h. An excess of stannous chloride (5 g) and EtOH (5 mL) was added to the resin. The resulting reaction mixture was stirred at 50°C for 8 h. At this time, the resin color changed from yellow to pale yellow. The cold resin was filtered and washed with water (4 × 20 mL). The resin was stirred in a solution of NaHCO_3_ (20% w/v, 20 mL), filtered, washed several times with water (2 × 20 mL), NaHCO_3_ solution (2 × 20 mL), MeOH (2 × 20 mL), and finally with CH_2_Cl_2_ (2 × 20 mL), and dried to give a yellow resin (1.8 g); *ν*_max⁡_/cm^−1^ 3360 (NH_2_).

#### 2.9.4. Preparation of 3-bromo-4-(4′-Resin-Supported benzylpiperazino)-1-formamide (**6**)

The resin-supported amine **5** (1 g) was suspended in dry DMF (10 mL) for 12 h before the addition of formic acid (5 mL). The reaction suspension was stirred and heated at 50°C for 2 h. the cooled reaction mixture was filtered and washed with water (4 × 10 mL) to remove the excess of formic acid. The resin was washed with NaHCO_3_ solution (30% w/v, 20 mL), MeOH (2 × 10 mL), and finally with CH_2_Cl_2_ (2 × 10 mL) to give derivatized resin **6** (1.2 g); *ν*_max⁡_/cm^−1^ 3362 cm^−1^(NH), 1721 cm^−1^(C=O).

#### 2.9.5. Preparation of Resin-Supported 7-bromo-6-piperazino-4-oxo-3-quinolone Carboxylic Acid (**7**)

3-Bromo-4-(4′-resin-supported benzylpiperazino)-1-formamide **(6)** (1 g) was suspended in dry DMF (10 mL) for 12 h. Phosphorus oxychloride (POCl_3_, 5 mL) was added to the suspended resin, and the mixture was stirred for 30 minutes at 25°C. A solution of methyl malonyl chloride (1.32 g, 9.6 mmoL) in POCl_3_ (2 mL) was gradually added to the reaction mixture. When the addition was completed, the temperature was gradually raised to 100°C for 24 h. After cooling, the reaction mixture was added gradually and carefully to ice (20 mL) then stirred for a further 20 minutes. The solution was basified using NaOH (10% w/v, 5 mL) and refluxed for a further 30 minutes. The resin was filtered and washed with water (2 × 10 mL), MeOH (2 × 10 mL), and finally with CH_2_Cl_2_ (2 × 10 mL) and dried *in vacuo* to constant weight (1.2 g); *ν*_max⁡_/cm^−1^ 1719 cm^−1^ (C=O).

### 2.10. Cleavage from the Resin

#### 2.10.1. Using the Hydrogenator

**General Method**

Resin-supported compound **4–7** (0.3 g) was placed in a hydrogenator vessel and suspended in dry CH_2_Cl_2_ (5 mL). Pd/C (0.05 g) was added to the resin suspension and the hydrogenation system was securely sealed. The reaction was carried out under 2 atm of hydrogen for 24 h. The reaction mixture was filtered, and the resin was washed several times with MeOH (4 × 5 mL); the resulting filtrates combined and the solvent was removed *in vacuo* to give a black residue (0.05 g). TLC showed a mixture of several spots, while the ^1^H NMR spectrum gave a complicated and noncharacterizable spectrum.

#### 2.10.2. Cleavage by Catalytic Transfer Hydrogenation (Hydrogenolysis)

**General Method**

The resin-supported compound **4–7** (0.3 g) was suspended in dry MeOH (10 mL). Cyclohexene (5 mL) and 20% Pd(OH)_2_ on carbon (1 : 3 catalyst substrate by weight) was added. The suspended mixture was stirred under dry nitrogen at reflux for 12–48 h; extra cyclohexene (10 mL) was added in two portions during this reaction time, and the reaction was monitored by TLC (CHCl_3_ : MeOH, 90 : 10). The reaction mixture was filtered through celite and washed with MeOH (3 × 10 mL). The combined filtrates were collected, dried over MgSO_4_, and concentrated to give a residue for characterization. None of the compounds **4–7** gave an acceptable cleavage product.

#### 2.10.3. Cleavage by Formation of a Solid-Supported Tertiary Amine Using Alkyl Halide

**General Method**

The compound on resin support **4–7** (0.3 g) was swollen with a mixture of DMF (5 mL), and an excess of MeI or EtI (3-4 mL) was added; the mixture was refluxed with slow stirring for 60 h. The resin was cross-washed with MeOH (5 × 10 mL), CH_2_Cl_2_ (5 × 10 mL), and diethyl ether (10 mL). The dry resin was swollen again with morpholine (4 mL) and heated at 110°C for 20–40 h and then washed with MeOH (2 × 3 mL), and the filtrate was evaporated. The resulting solid was partitioned between CH_2_Cl_2_ (5 mL) and aqueous sodium carbonate (10%, 5 mL). Organic layers were collected, dried, and concentrated. None of the expected cleavage products was obtained.

#### 2.10.4. Cleavage by Formation of a Solid-Supported Tertiary Amine Using *α*-Chloroethyl Chloroformate (ACE-Cl)

**General Method**

Compounds on the resin support (0.5 g) were first suspended in 1,2-dichloropropane (5 mL), followed by the addition of an excess of *α*-chloroethyl chloroformate (10 mL). The resulting suspension was stirred at room temperature for 48 h. The resin was filtered through a bed of silica gel, and the filtrate was then concentrated *in vacuo* until dryness. The residue dissolved in methanol and refluxed for 3 h. The solvent was removed to yield the secondary amines as their HCl salts.

3-Bromo-4-(4′-resin-supported benzylpiperazine)-1-nitrobenzene **(4)** (0.5 g) was swollen in 1,2-dichloropropane (5 mL) for 12 h, and ACE-Cl (10 mL) was then added. The resulting suspension was stirred at room temperature for 48 h and then treated as for the general method. The resulting black residue (0.3 g) was refluxed in ethanol for 3 h, and reaction was monitored by TLC. (CHCl_3_:petroleum ether (40–60), 60 : 40). The TLC showed a complicated mixture of spots; the major product at *R*_*f*_ = 0.34 was separated by preparative thin layer chromatography to give 3-bromo-4-ethoxy-1-nitrobenzene.

### 2.11. Preparation of N-(2-fluoro-5-nitrophenyl) piperazine (**13**) [15] the N,N-bis-(2-chloroethyl)ammonium chloride is very toxic and must be handled with care only in fuming hood

A mixture of 2-fluoro-5-nitroaniline (1 g, 6.4 mmoL) and *N,N-*bis-(2-chloroethyl)ammonium chloride (1.3 g, 7.0 mmoL) in diethylene glycol monomethyl ether (1 mL) was heated under dry nitrogen at 150°C for 24 h. The reaction was monitored by TLC (ethyl acetate : CHCl_3_, 80 : 20), product *R*_*f*_ = 0.42, the dark solid of *N-*(2-fluoro-5-nitrophenyl) piperazine 13 (0.87 g, 60%); mp 216-217°C; *ν*_max⁡_/cm^−1^ 3386 (NH), 1522 and 1346 (NO_2_); *δ*_H_ (300 MHz; DMSO-*d_6_*) [16] 3.26 (4H, m, CH_2_-3′ and CH_2_-5′), 3.40 (4H, m, CH_2_-2′ and CH_2_-6′), 7.49 (1H, dd, *J* = 9.0 Hz and 12 Hz, H-3), 7.85 (1H, dd, *J* = 3 and 9 Hz, H-6), 7.94 (1H, m, H-4), 9.59 (1H, br s, NH); *δ*_C_ (75 Hz; DMSO-*d*_6_) 43.0 (C-3′ and C-5′, 47.0 (C-2′ and C-6′), 115.2 (d, *J* = 5.3 Hz, C-6), 117.8 (d, *J* = 24 Hz, C-3), 119.2 (d, *J* = 10.5 Hz, C-4), 139.9 (d, *J* = 9.8 Hz, C-1), 144.9 (C-5), 158.8 (d, *J* = 257 Hz, C-2).

### 2.12. Solid Phase Reaction Using p-nitrophenyl Carbonate Wang Resin 14

#### 2.12.1. Reactions of N-(2-fluoro-5-nitrophenyl) piperazine with p-nitrophenyl Carbonate Wang Resin (**14**)

*p*-Nitrophenyl carbonate Wang resin **14 **(1 g, loading: 0.60–1.20 mmoL/g resin) was first suspended in dry DMF (5 mL) for 5 h, and *N*-(2-fluoro-5-nitrophenyl) piperazine **13** (1.6 g, 7.1 mmoL), and dry pyridine (2 mL) were then added to resin. The resulting suspension was then stirred and heated to 35°C for 24 h. After cooling to room temperature, the resin was filtered and washed with water (2 × 10 mL), methanol (3 × 10 mL) and CH_2_Cl_2_ (3 × 10 mL). The resin was dried under* vacuo* to give a brown resin (2.3 g). The residual product was verified by the complete disappearance of the characteristic carbonate resin band at 1760 cm^−1^; *ν*_max⁡_/cm^−1^ 1555 and 1316 (NO_2_), 1669 (C=O).

#### 2.12.2. Resin Cleavage [17]

The nitrocarbonate resin **15** (0.2 g) was suspended in trifluoroacetic acid (2 mL), dichloromethane (2 mL) and stirred at room temperature for 3 h. The cleavage reaction was monitored by TLC [(CH_2_Cl_2_ : MeOH, 80 : 20) on the solution, product *R*_*f*_ = 0.42]. The resin was filtered and washed with CH_2_Cl_2_ (4 × 20 mL), and the filtrate was collected and then extracted with NaHCO_3_ (10%, 4 × 20 mL). The CH_2_Cl_2_ layers were collected, washed with brine (2 × 20 mL), and dried over MgSO_4_. The solvent was removed under* vacuo* to give a yellow crystal of 13 (0.1 g).

#### 2.12.3. Reduction of N-(2-fluoro-5-nitrophenyl)piperazine-carbonate Wang Resin (**15**)

*N*-(2-fluoro-5-nitrophenyl)piperazine-carbonate Wang resin **15** (0.3 g) was suspended in anhydrous DMF (5 mL) and Et_3_N (2 mL). Anhydrous stannous chloride (1 g) and absolute ethanol (5 mL) were then added to the resin, and the reaction mixture was stirred at room temperature for 24 h (the color changed from deep yellow to light grey). The resin was filtered washed with methanol (20 mL), water (3 × 20 mL), methanol (3 × 10 mL), and CH_2_Cl_2_ (3 × 10 mL). The resin was dried to give the resin supported amine (0.34 g); *ν*_max⁡_/cm^−1^ 3401 and 3385 (NH_2_), 1672 (OC=O).

#### 2.12.4. Reaction of N-(2-fluoro-5-aminophenyl) piperazine-carbonate Wang Resin **16** with Ethyl Formate

*N*-(2-Fluoroaniline) piperazine-carbonate Wang resin (0.3 g) was suspended in dry DMF (5 mL) (the resin doubled in volume), under a positive flow of dry nitrogen, and ethyl formate was added (5 mL). The resulting mixture was stirred at 30°C for 24 h and, after cooling to room temperature, the resin was filtered off. TLC of the filtrate showed a spot at *R*_*f*_ = 0.32 (CHCl_3_ : MeOH, 96 : 4). The resin was washed with water (3 × 10 mL), methanol (3 × 10 mL), and CH_2_Cl_2_ (2 × 10 mL) to give, after drying, the corresponding formamide resin 16 (0.21 g); *ν*_max⁡_/cm^−1^ 3406 (NH) and 1685 (*N*-C=O), 1662 (OC=O).

### 2.13. Preparation of 1-(benzoylpiperazinyl)-2-fluoro-5-nitrobenzene (***19 ***)

*N*-(2-Fluoro-5-nitrophenyl)piperazine (**13)** (3 g, 13.3 mmoL) was dissolved in CHCl_3_ (20 mL), K_2_CO_3_ (5.5 g, 40 mml) and H_2_O (20 mL) were added to the above solution, and benzoyl chloride (3.73 g, 26.6 mmoL) was added gradually over 20 minutes. The reaction continued at 35°C for 1 hour. The organic layer was separated, washed with water (3 × 20 mL) and brine (30 mL), dried over MgSO_4_, and concentrated *in vacuo* to give a yellow shiny crystal of **19** (4 g, 92%); mp 108-109°C; (Calc. for C_18_H_18_FN_3_O_2_: C, 66.0; H, 5.5; N, 12.8. Found: C, 66.1; H, 5.5; N, 12.7); *ν*_max⁡_/cm^−1^ 1694 (CO-N), 1508 (NO_2_), 1347 (NO_2_); *δ*_H_ (300 MHz; DMSO-*d*_6_) 2.94 (4H, s, CH22′, CH2-6′), 3.49 (2H, s, CH2-3′), 3.71 (2H, s, CH2-5′), 7.46 (6H, m, Ph + H-3), 7.81 (1H, dd, *J* = 3 and 7.5 Hz, H-6), 7.92 (1H, m, H-4); *δ*_C_ (75 MHz,; DMSO-*d*_6_) 41.3 (C-5′), 46.9 (C-3′), 49.6 (C-2′ and C-6′), 115.2 (d, *J* = 5.2 Hz, C-6), 117.7 (d, *J* = 23.0 Hz, C-3), 118.8 (d, *J* = 9.5 Hz, C-4), 128.9 (Ph), 135.9 (C-8), 140.7 (d, *J* = 10 Hz, C-1), 144.9 (C-5), 158.9 (d, *J* = 254 Hz, C-2), 169.6 (C=O).

### 2.14. Preparation of 1-(benzoylpiperazinyl)-2-fluoro-aniline (**20**) [14]

1-(benzoylpiperazinyl)-2-fluoroaniline (**20)** (2.14 g, 78%); mp 89-90°C; (Calc. for C_17_H_18_FN_3_O : C, 68.2; H, 6.1; N, 14.0. Found: C, 68.2; H, 6.05; N, 14.0); *ν*_max⁡_/cm^−1^ 3450 and 3350 (NH_2_), 1724 (CO-N); *δ*_H_ (300 MHz; DMSO-*d*_6_) 2.94 (4H, s, CH_2_-2′ and CH_2_-6′), 3.48 (2H, s, CH_2_-3′), 3.73 (2H, s, CH_2_-5′), 4.84 (2H, br s, NH_2_), 6.13 (1H, m, H-4), 6.25 (1H, dd, *J* = 2.4 and 7.5 Hz, H-6), 6.77 (1H, dd, *J* = 8.7 and 12.6 Hz, H-3), 7.45 (5H, m, Ph); *δ*_C_ (75 MHz; DMSO-*d*_6_) 50.17 (2CH_2_), 50.22 (2CH_2_), 105.5 (C-6), 107.6 (d, *J* = 7.1 Hz, C-4), 116.3 (d, *J* = 21.2 Hz, C-3), 128.9 (Ph), 140.0 (d, *J* = 9.5 Hz, C-1), 145.9 (C-5), 147.7 (d, *J* = 230 Hz, C-2), 169.5 (C=O).

### 2.15. Preparation of 1-(benzoylpiperazinyl)-2-fluoro-formanilide (**21**)

Formic acid (5 mL) was added to 1-(benzoylpiperazin-1-yl)-2-fluoroaniline (**20)** (1 g, 3.34 mmoL); the resulting solution was heated at 70°C for 2 h. The cooled reaction mixture was added to cooled water (100 mL), extracted with CHCl_3_ (4 × 20 mL) and brine (30 mL), and dried over MgSO_4_. The resulting white solid was purified by column chromatography (CHCl_3_ : MeOH, 97 : 3) to give white crystals of **21** (0.62 g, 56%); mp 189–191°C; (Calc. for C_18_H_18_FN_3_O_2_: C, 66.0; H, 5.5; N, 12.8. Found: C, 66.0; H, 5.5; N, 12.8); *ν*_max⁡_/cm^−1^ 3080 (NH) 1684 (NH-CHO), 1620 (CO-Ph); *δ*_H_ (300 MHz; DMSO-*d*_6_) 3.02 (4H, s, H-2′, H-6′), 3.55 (2H, s, H-3′), 3.74 (2H, s, H-5′), 7.11 (1H, dd, *J* = 8.7 and *J* = 12.0 Hz, H-3), 7.19 (1H, ddd, *J* = 8.7, 2.7 and 1.5 Hz, H-4), 7.35 (1H, dd, *J* = 2.7 and *J* = 5.4 Hz, H-6), 7.46 (5H, m, CO-Ph), 8.25 (1H, d, *J* = 1.8 Hz, CHO), 10.12 (1H, br s, NH); *δ*_C_ (75 MHz; DMSO-*d*_6_) 50.2 and 50.22 (piperazine-C), 111.1 (C-6), 113.7 (d, *J* = 7.5 Hz, C-4), 116.5 (d, *J* = 21.0 Hz, C-3), 128.9 (Ph), 136.3 (C-5), 140.0 (d, *J* = 9.0 Hz, C-1), 153.1 (d, *J* = 240 Hz, C-2), 159.9 (CHO), 169.6 (C=O).

### 2.16. Vilsmeier Reaction of 3-nitro-4-fluoroformanilide

*Preparation of N*,*N*′*-Bis-(4-fluoro-3-nitrophenyl)oxala-mide **23**.*

Under anhydrous conditions, 3-chloro-4-fluoroforma-nilide (2 g, 10.86 mmoL) was dissolved in dry CHCl_3_ (20 mL), then (COCl)_2_ (2 mL) was added gradually over 30 minutes, (a vigorous reaction was observed). The resulting reaction mixture was heated to 40°C for 30 minutes. The reaction flask was removed from the oil bath; methyl malonyl chloride (1.78 g, 13.03 mmoL) in CHCl_3_ (2 mL) was added gradually to the Vilsmeier reagent over 30 min. The reaction was continued at 40°C for 3 h until the TLC of the reaction showed a complete consumption of the starting formanilide, with the formation of a new product above the starting compound, [(CHCl_3_ : MeOH, 95 : 5) *R*_*f*_ = 0.56]. The reaction mixture was concentrated *in vacuo*, followed by the addition of cooled water (20 mL), and stirred for 30 minutes. The resulting yellow solid was collected by filtration, washed with water, and recrystallized from CHCl_3_ to give yellow crystals of *N*, *N*′-Bis-(4-fluoro-3-nitrophenyl)oxalamide **(23)** (0.62 g, 16%); mp 109–111°C; (Calc. for C_14_H_8_F_2_N_4_O_6_: C, 45.9; H, 2.2; N, 15.3. Found: C, 45.9; H, 2.2; N, 15.3); *ν*_max⁡_/cm^−1^ 3275 (NH), 1673 (NCO); ^1^H NMR *δ*_H_ (300 MHz; DMSO-*d*_6_) 7.64 (2H, t, *J* = 9.0 Hz, H-5 and H-5′), 8.23 (2H, m, H-6 and H-6′), 8.80 (2H, dd, *J* = 0.8 and 1.5 Hz, H-2 and H-2′), 11.40 (2H, s, 2NH); *δ*_C_ (75 MHz; CDCl_3_) 120.1 (C-2 and C-2′), 121.7 (d, *J* = 22.5 Hz, C-5 and C-5′), 130.9 (d, *J* = 7.5 Hz, C-6 and C-6′), 137.2 (C-1 and C-1′), 139.1 (d, *J* = 7.5 Hz, C-3 and C-3′), 154.1 (d, *J* = 262.5 Hz, C-4 and C-4′), 161.2 (C=O).

### 2.17. Vilsmeier Reaction on 3-chloro-4-fluoroformanilide and Preparation of *N*,*N*′-Bis-(3-chloro-4-fluorophenyl)malon-amide (**24**)

Under anhydrous conditions, 3-chloro-4-fluoroformanilide (2 g, 12.98 mmoL) was dissolved in CHCl_3_ (20 mL). Oxalyl chloride (2 mL) was added gradually over 30 min. (vigorous reaction). The resulting reaction mixture was heated to 40°C for 30 minutes. Methyl malonyl chloride (2.13 g, 15.57 mmoL) was added gradually to the cooled reaction mixture over 30 minutes. When addition was complete, the reaction was continued at 40°C for 3 h until TLC showed a complete consumption of the starting formanilide with formation of a new product above the starting compound [(CHCl_3_ : MeOH, 97 : 3) *R*_*f*_ = 0.53]. The reaction mixture was concentrated *in vacuo*; cold water (20 mL) was then added and the mixture stirred for 30 minutes. The resulting yellow solid was collected by filtration, washed with water, and purified by column chromatography (CHCl_3_). The solid was recrystallized from CHCl_3_ to give shiny needle-like crystals of compound **24** *N*, *N*′-Bis-(3-chloro-4-fluorophenyl)malon-amide (0.72 g, 18%); mp 201-202°C; (Calc. for C_15_H_10_Cl_2_ F_2_N_2_O_2_: C, 50.2; H, 2.8; N, 7.8. Found: C, 50.2; H, 2.8; N, 7.8); *ν*_max⁡_/cm^−1^ 3281 (br, NH), 1677 (C=O), 1497 (NH), 811 (Cl-C=O); *δ*_H_ (300 MHz; DMSO-*d*_6_) 7.38 (2H, t, *J* = 9.0 Hz, H-5 and H-5′), 7.48 (2H, ddd, *J* = 2.4, 4.5 and 9.0 Hz, H-6 and H-6′), 7.93 (2H, dd, *J* = 2.4 and 2.7 Hz, H-2 and H-2′), 10.39 (2H, s, 2NH); *δ*_C_ (75 MHz,; DMSO-*d*_6_) 46.3 (CH_2_), 117.5 (d, *J* = 21.7 Hz, C-5 and C-5′), 119.7 (d, *J* = 18 Hz, C-3 and C-3′), 119.9 (d, *J* = 7.5 Hz, C-6 and C-6′), 121.0 (C-2 and C-2′), 136.6 (C-1 and C-1′), 153.7 (d, *J* = 240 Hz, C-4 and C-4′), 165.9 (C=O).

### 2.18. Physicochemical Studies

The physicochemical studies include the lipophilicity, Fourier transforms infrared spectroscopy, and the thermal stability of highly bioactive compounds. The thermal behaviors for the bioactive compound **12** was investigated by thermogravimetric technique and indicated by the TGD peaks at 177 and 270°C (Figures 3 and 4). The highly bioactive pure tested compounds were also determined like melting point, water solubility and pKa values.

### 2.19. Antimicrobial Assay

Some synthesized compounds, **19**, **20**, **21**, **9**, **11**, **23**, and **12,** were evaluated for their antimicrobial effects by Agar diffusion disk method [18] using Nutrient Agar, MacConkey Agar, and Sabouraud Dextrose Agar. The potentialities of these compounds were estimated against some important and representative microbes like Gram + ve: *Bacillus Subtilis* (B.S.); *Staphylococcus Aureus* (S.A.), Gram −ve: *Escherichia Coli *(E.C.); *Klebsiella Pneumonia (*K.P.), and Fungi: *Candida Albicans* (C.A.); *Aspergillus Funigates* (A.F.). The presterilized filter paper disks (6 mm diameter) were impregnated with 30, 40, and 50 *μ*g of the compound and dissolved in DMF as solvent, which has no effect on either bacteria or fungi. These disks were implanted on different sets of agar plates containing the microbes. The agar plates were then incubated for 24 hours at 37°C for bacteria and for 7 days at 28°C for fungi. Nalidixic acid and nystain were used as reference antibiotics. 

In addition, similar antimicrobial assay was performed for the biologically highly active compounds **20, 21, 11, 23,** and **12** after exposure of the Petridishes containing microorganisms and the test compounds to UV light (*λ*366 nm) for 3 hours before the incubation.

## 3. Results and Discussion

### 3.1. Synthesis of Novel Norfloxacin Analogues

In the present study, novel norfloxacin analogues were synthesized using basically the Vilsmeier method with some modifications. The 7-bromo-6-N-benzyl piperazinyl-4-oxoquinoline-3-carboxylic acid **(12)** was isolated at high temperature (mention the temperature). On the other hand, bis-compounds *N*, *N*′-bis-(4-fluoro-3-nitrophenyl)-oxalamide and *N*, *N*′*-*bis-(3-chloro-4-fluorophenyl)- malonamide **(22)** and **(23)** were obtained under reveres Vilsmeier approach using the modified method of commercially available Merrifield resin **14**, which was modified by introduction of spacer with free hydroxyl group to enhance the activity of the substrates bound to the polymer. Besides the determination of their physiochemical properties, these compounds were evaluated for use in vitiligo and as antimicrobial agents. 

**Scheme 4:**
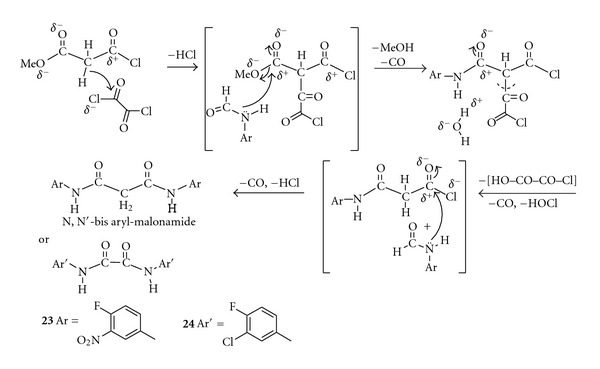
A possible formation of *N*, *N*′-bis-Aryl malonamide instead of norfloxacin analogues.

Isolation of two novel *N*, *N*-bis-(aryl) compounds **23**,** 24** instead of norfloxacin analogue targets could be due to a type of interaction between oxalyl chloride with methyl malonyl chloride followed by monoacylation of anilidimide which hinders the formation of norfloxacin analogues via a second interaction with other anilidimide molecule (Scheme 4). Recently, nonfluorinated *N*, *N*-bis-aryl derivative was reported as an HIV-1 integrase inhibition [19].

### 3.2. Physiochemical Properties

#### 3.2.1. Lipophilicity

**Scheme 5:**
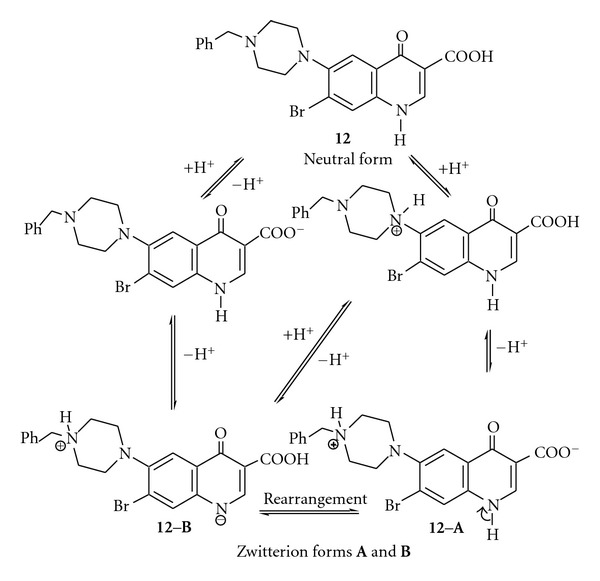


The lipophilic and Zwitterionic form of the obtained compounds, as well as steric and electronic effects or charge density, plays an important role for chemical and biocidal activities. *N*-Mannich base functional group can increase the lipophilicity of the tested compounds, for example, **12** at physicobiological pH values by decreasing their protonation resulting in the enhancement of absorption through biomembranes. It is clear that the neutral species of haloquinolones are more lipophilic than Zwitter ionic form. In addition, steric and electronic effects or molecular charge density can affect lipophilicity (Scheme 5).

#### 3.2.2. Fourier Transforms Infrared Spectroscopy

**Figure 1:**
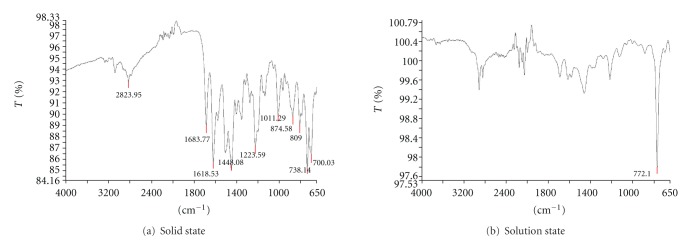
FT-IR spectrum of 12 in (a) solid and (b) solution states for compound **12**.

**Figure 2:**
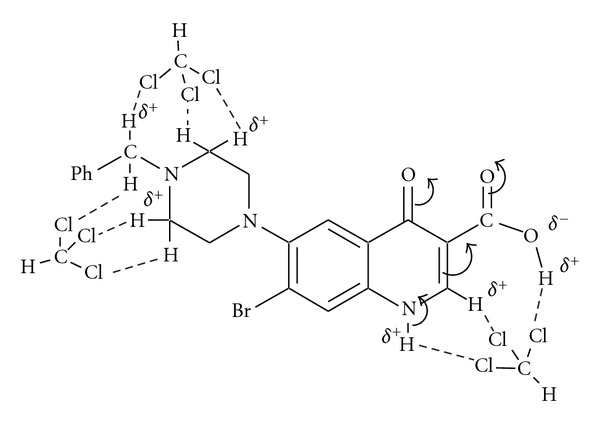
The interaction between compound **12** with CHCl_3_.

**Figure 3:**
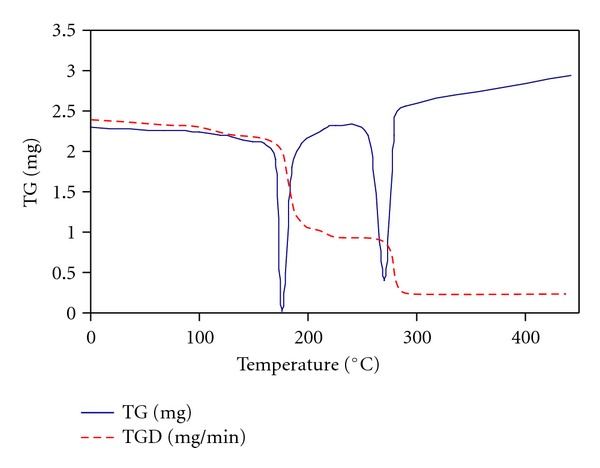
TG and TGD for compound **12**.

**Figure 4:**
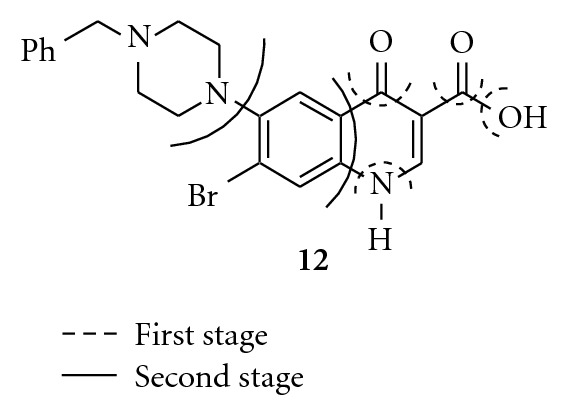
Suggested decomposition stages for compound **12**.

Generally, Fourier transforms infrared spectroscopy (FT-IR) studies of the obtained compounds in both the solid and solution (CHCl_3_) states showed lack of some characteristic bands in the solution state, for example, compound **12 **(Figure 2). This effect may be due to a type of intramolecular and/ or intermolecular H-bonding between functional group of the tested compounds and a functional group in the solvent used, which possibly act similarly to the functional groups of the organisms leading to inhibition of their vital activities and death. The results of the Fourier transform infrared spectroscopy are given in Figure 1.

#### 3.2.3. Other Physicochemical Properties of Highly Bioactive Compounds

The physicochemical properties of highly bioactive pure tested compounds are demonstrated as follows.

- *Melting Points*. They differ according to the type of solvent from which crystals are obtained, for example, compound 20 had approximately 87°C for pure crystallized from cyclohexane, and 90°C from chloroform.
- *Solubility in Water*. Pure compound** 20**, for example, gave approximately, 200 *μ*g/L while compound **23 **showed 350 *μ*g/L at 20°C.
- *Pka*. Pure tested compounds at pH 5.7 and 9 at 24°C showed different types of protons, in quinolone the –COOH and NH, while in the formylamino derivative, –COOH, –CHO, and NH. This data indicated that tested compounds **20, 21, 11, 23, **and **12 **have a very low rate of hydrolysis because of its stability in suspension concentration under normal conditions Table 4.

### 3.3. Antimicrobial Assay

The potentialities of the tested compounds **19**, **20**, **21**, **9**, **11**, **23**, and **12** are given in Tables 1, 2 and 3.

### 3.4. Photochemical Probe Agents

Vitiligo is an acquired disorder characterized by patchy progressive depigmentation of the skin. It affects about 2% of world population. Vitiligo occurs equally in both sexes and has no age limits. It may be presented as a single path, which may be progressing or static for a long time and suddenly starts progressing or multiple patches, which are slowly progressing or stationary indefinitely. These depigmented molecules sometimes spontaneously pigment and depigment again and are often symmetrical and are called as vitiligo vulgarize. The etiology of nonsymmetrical Vitiligo, namely, segment vitiligo, is entirely different from symmetrical vitiligo. Often the exposed areas of the skin and areas around orifices of the body are depigmented rather than other areas [20]. The melanocytes successfully treated vitiligo patients by PUVA therapy [21]. Increasing use of PUVA-8MP could be responsible for a type of skin cancer [22]. Thus, some antibiotics like nalidixic acid and Nystatin are now used to control the vitiligo symptoms.

## Figures and Tables

**Table 1:** The preliminary screening of antimicrobial activity of the new synthesized compounds.

Compounds/DMF 50 *μ*g/ml disk	Microorganisms/Inhibition zone (mm)					
	Gram +ve bacteria^a^		Gram − ve bacteria^b^		Fungi^c^	
	*B.S.*	*S.A*	*E.C.*	*K.P.*	*C.A.*	A.F.
**19**	9	8	8	10	10	6
**20**	18	16	15	14	10	6
**21**	16	16	14	14	8	6
**9**	8	8	7	11	9	6
**11**	13	11	12	13	9	6
**23**	15	14	15	13	10	6
**12**	20	19	18	17	10	10
Ny.	6	6	6	10	10	32
Na.	32	30	30	22	6	6

Ny: nystatin, manufactured by Pasteur Lab., Egypt. NS 100 units (100 *μ*g/disk).

Na: nalidixic acid, 30 *μ*g/disk, Bioanalize, Egypt.

^a^ *Bacillus Subtilis (B.S.) *and* Stphylacoccus Aureus (S.A.);* ^b^ *Escherichia Coli (E.C.) *and* Klebsiella Pneumonia (K.P.);* ^c^ *Candida Albicans (C.A.) *and* Aspergillus Funigates (A.F.).*

**Table 2:** MIC of the active biological compounds towards bacteria.

Compd. No.	Inhibition Zones (*μ*g /mm)											
	*B.S.*			*S.A.*			*E.C.*			*K.P.*		
	50	40	30	50	40	30	50	40	30	50	40	30

**20**	18	12	6	16	14	10	15	12	10	14	12	8
**21**	16	14	6	16	12	10	14	13	10	14	11	9
**11**	13	10	6	11	10	10	12	10	6	13	11	10
**23**	15	12	6	14	12	10	15	12	6	13	11	9
**12**	20	18	15	19	16	10	18	16	12	17	14	12

**Table 3:** Preliminary screening using UV (*λ*366 nm) light, conc. 50 *μ*g/disk.

Compd. No.	Microorganisms (inhibition zones in mm)					
	+ve bacteria		− ve bacteria		Fungi	
	*B.S.*	*S.A.*	*E.C*	*K.P.*	*C.A.*	A.F.

**20**	18	16	15	14	No change	No change
**21**	18	17	16	18	No change	No change
**11**	14	14	12	14	No change	No change
**23**	17	16	17	16	No change	No change
**12**	24	21	21	21	No change	No change

**Table 4:** Various physicochemical properties of highly bioactive compounds.

Compd. No.	MIC at 30 *μ*g/disc				Mol. Mass	Melting point		Solubility^c^ in water (20°C), *μ*g/L
	*B.S*	*S.A*	*E.C*	*K.P*		CHCl_3_^a^	Cyclo-Hexane^b^	

**20**	6	10	10	8	299	90	87	200
**21**	6	10	10	9	327	191	186	300
**11**	6	10	6	10	374	74	70	90
**23**	6	10	6	9	366	111	107	350
**12**	15	10	12	12	442	285	—	400
Ny	6	6	6	6	—	—	—	—
Na	32	30	30	32	—	—	—	—

^a^Crystals cyclisation from CHCl_3_; ^b^Crystals cyclisation from cyclohexane; ^c^hydrolysis characteristics at pH 5.7 and 9 and at 24°C. The tested compounds have a very low rate of hydrolysis, which is considered stable in suspension concentrations under normal condition.
